# Anti-androgenic therapy with finasteride in patients with chronic heart failure - a retrospective propensity score based analysis

**DOI:** 10.1038/s41598-019-46640-8

**Published:** 2019-07-12

**Authors:** Badder Kattih, Lukas Simon Elling, Christel Weiss, Marieke Bea, Carolin Zwadlo, Udo Bavendiek, Johann Bauersachs, Joerg Heineke

**Affiliations:** 1 Department of Cardiology and Angiology, Hannover Medical School, Carl-Neuberg Street 1, 30625 Hannover, Germany; 2 Department of Cardiovascular Research, European Center for Angioscience (ECAS), Medical Faculty Mannheim of Heidelberg University, University Medical Centre Mannheim, Ludolf-Krehl Street 7-11, 68167 Mannheim, Germany; 3 Department for Medical Statistics and Biomathematics, Medical Faculty Mannheim of Heidelberg University, University Medical Centre Mannheim, Ludolf-Krehl Street 9-13, 68167 Mannheim, Germany

**Keywords:** Cardiac hypertrophy, Heart failure, Translational research

## Abstract

Sex hormones influence the prevalence and the outcome of heart diseases. The conversion of testosterone to its more active metabolite dihydrotestosterone drives cardiac growth and dysfunction, while inhibition of this step by the anti-androgenic drug finasteride counteracts these pathological processes in preclinical models. In this retrospective, observational study, we aim to investigate whether finasteride, which is in clinical use mainly for prostate disease, might ameliorate cardiac hypertrophy and heart failure in patients. Retrospective chart review of 1041 medical cases with heart failure between 1995 and 2015 was conducted. Stratification was performed by concomitant prostate treatment status (tamsulosin versus finasteride). A propensity score analysis yielded a total of 328 matched medical cases without residual differences in the baseline patient characteristics. In this propensity score matched samples, anti-androgenic therapy with finasteride was associated with significantly reduced left ventricular hypertrophy (interventricular septal thickness 13.3 ± 2.4 mm control vs. 12.6 ± 2.1 mm finasteride group (p = 0.029); estimated average treatment effects on the treated: −0.7 mm, 95% CI mean difference −1.3 to −0.1). In this retrospective analysis anti-androgenic therapy with finasteride for prostate disease was associated with attenuated cardiac hypertrophy in patients with heart failure. Therefore, our data encourage further analysis of this approach in larger heart failure patient cohorts.

## Introduction

Despite recent therapeutic advances, mortality rates in patients with prior hospitalizations for heart failure remain higher than for many malignancies^1^. The progression of heart failure is driven by maladaptive myocardial remodeling processes (i.e. mainly left ventricular hypertrophy)^2,3^.

While current standard medical therapy targets predominantly neurohormonal activation, increasing evidence points towards additional, deleterious pathways driving the progression of heart failure. Sex hormones, for instance, might play a role given the fact that premenopausal women with heart failure have a better prognosis compared to men, while after menopause this phenomenon is at least partially reversed^4–6^. Although estrogen has been deemed cardioprotective, large studies failed to demonstrate beneficial effects of hormone replacement therapy in postmenopausal women^7–9^. Therefore, the rise in cardiovascular mortality in women after menopause was suggested to occur as consequence of increased ovarian production of testosterone^10^. Indeed, testosterone and especially the more active dihydrotestosterone or anabolic androgenic steroids trigger cardiac hypertrophy in isolated cardiomyocytes, mice and human weight lifters, respectively^11–15^. We recently showed in mice that finasteride - a drug commonly used in patients to treat prostate disease - potently reverses pathological cardiac hypertrophy and left ventricular dysfunction via inhibition of the enzyme 5α-reductase, which catalyzes the conversion of testosterone to the about 10-fold more active dihydrotestosterone^16^. Insights into possible treatment effects of finasteride in human heart failure, however, are currently lacking and it remains unclear how anti-androgenic therapy should be conducted.

In this retrospective study, we aim to investigate whether treatment with finasteride might have beneficial effects on the development of pathological hypertrophy in patients suffering from heart failure.

## Results

**Figure 1:**
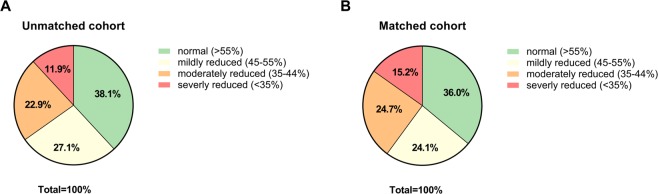
Left ventricular function based on cardiac imaging: (**A)** In the unmatched study cohort (**B)** In the matched study cohort. Raw values are provided in Table S4 in the Data Supplement.

We analyzed 1041 medical cases with heart failure (HFrEF or HFpEF were eligible), whereby 868 of these cases were in the control group (tamsulosin only) and 173 in the treatment group (finasteride with or without tamsulosin). The baseline characteristics of both groups are summarized in Table 1 (and Table S1 in the Data Supplement). In the unmatched study cohort, 11.9% had a severely reduced LV function; in 22.9%, the LVEF was moderately decreased and in 27.1% mildly decreased, and 38.1% had an ejection fraction >55% (Fig. 1A). Patients in the treatment group were on average about 2 years older compared to those in the control group (p = 0.006) and had a significant higher prevalence of cardiovascular risk factors (history of smoking 45.7% vs. 33.5% (p = 0.002) and hypertension 83.8% vs. 75.6% (p = 0.019)) and a lower body mass index 25.8 vs. 27.1 kg/m² (p < 0.001). Aspirin and statins were more frequently prescribed in the control group (55.9% vs. 46.2% (p = 0.020) and 70.4% vs. 58.4% (p = 0.002)) as depicted in Table 1. Additionally, a higher NYHA status and percentage of patients with acute cardiac decompensation was observed in the treatment group in the unmatched cohort (Table S1 in the Data Supplement). Notably, the use of guideline-directed heart failure therapy (including ACE inhibitors, angiotensin receptor blockers, beta-blockers, mineralocorticoid receptor-antagonists) did not differ significantly between the finasteride treatment and the control group.

**Table 1:** Baseline characteristics pre- and post-propensity score matching*.

	unmatched cohort (total n = 1041)				matched cohort (total n = 328)			
	Tamsulosin	Finasteride	p value	SMD	Tamsulosin	Finasteride	p value	SMD
	(total n = 868)	(total n = 173)			(total n = 164)	(total n = 164)		
Age [yr]	74.1 ± 8.0	75.9 ± 6.7	0.006	0.20	75.9 ± 7.7	75.8 ± 6.7	0.862	−0.01
Body mass index [kg/m²]	27.1 ± 3.8	25.8 ± 3.8	<0.001	−0.26	25.9 ± 3.6	25.9 ± 3.8	0.746	0.02
Systolic blood pressure [mmHg]	132.4 ± 46.3	129.1 ± 19.6	0.352	−0.09	128.6 ± 21.6	129.5 ± 18.9	0.607	0.04
Diastolic blood pressure [mmHg]	74.5 ± 11.7	74.1 ± 12.3	0.576	−0.03	75.0 ± 11.9	73.9 ± 11.6	0.346	−0.08
Heart rate [beats per min]	72.6 ± 17.1	70.2 ± 13.3	0.209	−0.14	68.3 ± 15.7	70.1 ± 13.5	0.152	0.10
COPD	129/868 (14.9)	34/173 (19.7)	0.113	0.10	33/164 (20.1)	32/164 (19.5)	0.890	−0.01
History of smoking	291/868 (33.5)	79/173 (45.7)	0.002	0.20	72/164 (43.9)	74/164 (45.1)	0.824	0.02
Hypertension	656/868 (75.6)	145/173 (83.8)	0.019	0.17	134/164 (81.7)	137/164 (83.5)	0.662	0.04
Hyperlipidemia	529/868 (60.9)	117/173 (67.6)	0.098	0.12	108/164 (65.9)	109/164 (66.5)	0.907	0.01
Diabetes mellitus	240/868 (27.6)	51/173 (29.5)	0.624	0.03	42/164 (25.6)	48/164 (29.3)	0.458	0.07
Aspirin	485/868 (55.9)	80/173 (46.2)	0.020	−0.16	80/164 (48.8)	77/164 (46.9)	0.740	−0.03
ACEi/ARBs	670/868 (77.2)	138/173 (79.8)	0.457	0.05	134/164 (81.7)	132/164 (80.5)	0.778	−0.03
Beta - blockers	661/868 (76.2)	124/173 (71.7)	0.212	−0.08	120/164 (73.2)	119/164 (72.6)	0.901	−0.01
MR - antagonists	159/868 (18.3)	35/173 (20.2)	0.555	0.04	29/164 (17.7)	33/164 (20.1)	0.573	0.05
Statins	611/868 (70.4)	101/173 (58.4)	0.002	−0.20	101/164 (61.6)	99/164 (60.4)	0.821	−0.02
**Prostate disease**								
Benign prostatic hyperplasia	836/868 (96.3)	169/173 (97.7)	<0.001	0.07	154/164 (93.9)	160/164 (97.6)	0.005	0.16
Prostate cancer	32/868 (3.7)	1/173 (0.6)		−0.20	10/164 (6.1)	1/164 (0.6)		−0.29
N/A	0/868 (0.0)	3/173 (1.7)		0.13	0/164 (0.0)	3/164 (1.8)		0.14

*More details regarding characteristics of patients are provided in Table S1 in the Data Supplement. Values are expressed as mean ± SD or n/total n (%). Abbreviation: ACEi = angiotensin converting enzyme inhibitor, ARB = angiotensin receptor blocker, COPD = Chronic obstructive pulmonary disease, N/A = not available, SMD = standard mean difference.

**Figure 2:**
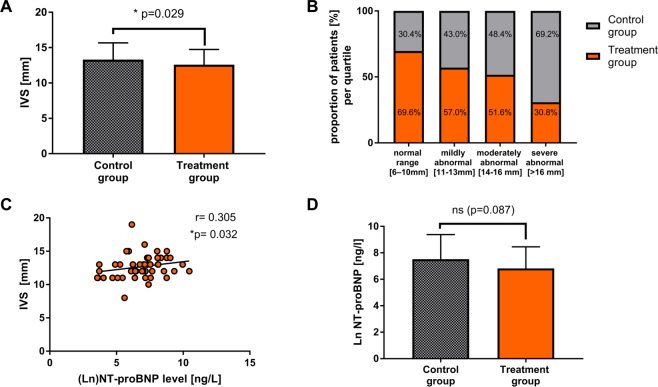
(**A)** Septal thickness (IVS) is significantly reduced in the finasteride group compared to the control group. (**B)** The graph reveals that less finasteride treated patients were found the higher the severity of cardiac hypertrophy (IVS) was. (**C)** NT-proBNP level are significantly correlated with severity of septal thickness indicating an association between severity of cardiac hypertrophy and the ventricular filling pressure in the study cohort. (**D)** Finasteride treated patients display a trend towards lower NT-proBNP level. Raw values are provided in Tables S2 and S3 in the Data Supplement.

Next, to get a better adjustment of patient characteristics between the treatment and control group, we employed a propensity score method. Matching the patients in both groups on the logit of the propensity score revealed 164 medical cases in each group resulting in an overall balance of baseline covariates (with the exception of prostate disease) for the propensity score matched population, while NYHA status remained different with more finasteride patients in the NYHA III class (Table 1 and Fig. S1 in the Data Supplement). In the matched study cohort, 15.2% had a severely reduced LV function; in 24.7%, the LVEF was moderately decreased and in 24.1% mildly decreased, and 36.0% had an ejection fraction >55% (Fig. 1B). The percentage of patients with acute cardiac decompensation or diastolic dysfunction did not differ significantly between both groups (Table S1 in the Data Supplement). Clinical outcomes of the total and propensity score matched cohorts are shown in Table 2 and Table S2 in the Data Supplement. After propensity score matching, the mean interventricular septal thickness was significantly reduced in the treatment group (13.3 ± 2.4 mm control group vs. 12.6 ± 2.1 mm finasteride group, p = 0.029), so that the estimated average treatment effects on the treated (ATT) were −0.7 mm (95% CI −1.3 to −0.1), indicating an anti-hypertrophic effect in the finasteride group (Fig. 2A). There were no significant differences in other outcome parameters (Table 2 and Table S2 in the Data Supplement). The anti-hypertrophic effects of finasteride were also revealed by an analysis showing that the proportion of finasteride treated patients decreased with increasing severity levels of the septal thickness (Fig. 2B). Interestingly, NT-proBNP levels significantly correlated with the severity of interventricular septal thickness in the study cohort (Fig. 2C) and tended to be lower in the finasteride treated group compared to the control group (Fig. 2D), while statistical significance was reached solely in subgroup analysis of acutely decompensated patients despite low number of values (Table S5 in the Data Supplement).

**Table 2:** Outcome parameters pre- and post- propensity score matching*.

	unmatched cohort (total n = 1041)				matched cohort (total n = 328)			
	control group (total n = 868)	treatment group (total n = 173)	p value	ATT (95% confidence intervall)	control group (total n = 164)	treatment group (total n = 164)	p value	ATT (95% confidence intervall)
(Ln)NT-proBNP [ng/l]	7.1 ± 1.7 (n = 168)	6.9 ± 1.6 (n = 41)	0.588	−0.2 (−0.7 to 0.4)	7.5 ± 1.9 (n = 34)	6.8 ± 1.6 (n = 39)	0.087	−0.7 (−1.5 to 0.1)
Ejection fraction [%]	47.8 ± 14.7 (n = 354)	46.2 ± 13.4 (n = 70)	0.400	−1.6 (−5.3 to 2.2)	46.8 ± 16.5 (n = 68)	45.6 ± 13.4 (n = 66)	0.641	−1.2 (−6.4 to 3.9)
IVS [mm]	12.9 ± 2.3 (n = 464)	12.6 ± 2.2 (n = 122)	0.385	−0.2 (−0.7 to 0.2)	13.3 ± 2.4 (n = 93)	12.6 ± 2.1 (n = 114)	0.029	−0.7 (−1.3 to −0.1)
LVEDD [mm]	53.6 ± 8.5 (n = 540)	54.4 ± 8.1 (n = 141)	0.164	0.8 (−0.8 to 2.4)	52.6 ± 9.0 (n = 109)	54.6 ± 8.0 (132)	0.131	2.1 (−0.7 to 4.2)
LA-PLAX [mm]	45.2 ± 7.4 (n = 168)	44.9 ± 7.8 (n = 140)	0.463	−0.2 (−1.6 to 1.2)	45.0 ± 7.6 (n = 96)	44.8 ± 7.8 (n = 131)	0.554	−0.2 (−2.2 to 1.8)
QRS duration [ms]	120.0 ± 69.6 (n = 391)	123.1 ± 31.7 (n = 145)	0.010	3.0 (−8.7 to 14.8)	117.4 ± 27.7 (n = 72)	123.5 ± 31.9 (n = 137)	0.137	6.2 (−2.6 to 14.9)
QT duration [ms]	411.8 ± 49.7 (n = 244)	413.8 ± 58.8 (n = 135)	0.794	1.9 (−9.3 to 13.1)	420.5 ± 40.8 (n = 45)	417.2 ± 52.2 (n = 127)	0.396	−3.3 (−20.2 to 13.7)
QTc duration [ms]	448.7 ± 190.6 (n = 251)	443.9 ± 54.8 (n = 140)	0.249	−4.8 (−37.2 to 27.6)	445.2 ± 45.3 (n = 47)	446.5 ± 48.6 (n = 132)	0.858	1.4 (−14.6 to 17.4)

*LV dimensions normalized to BSA are provided in Table S2 in the Data Supplement. Abbreviation: ATT = average treatment effect on the treated, IVS = Interventricular septum, LA-PLAX = Left atrium in parasternal long axis, LVEDD = Left ventricular enddiastolic diameter.

## Discussion

In this retrospective study, we found that finasteride treatment for prostate disease in patients with heart failure is associated with reduced cardiac hypertrophy. Although we cannot infer causality from this retrospective analysis, the average treatment effects on the treated revealed that patients receiving anti-androgenic therapy with finasteride displayed significantly reduced septal thickness after achieving a balanced distribution of baseline characteristics between control and treatment group based on propensity score matching. In addition, with increasing severity of the septal thickness the proportion of finasteride treated patients decreased. Notably, NT-proBNP level significantly correlated with severity of septal thickness and tended to be lower in the finasteride treated group compared to the control group, while in both groups the fraction of acutely decompensated heart failure patients did not differ significantly. These results point towards an anti-hypertrophic benefit of finasteride treatment in patients with heart failure^17^, which might be of clinical importance since left ventricular hypertrophy is not only a common precursor of heart failure^18–20^, but is also directly associated with diastolic and systolic heart failure^21–24^. In turn, regression of left ventricular hypertrophy was previously shown to improve left ventricular dysfunction and cardiovascular events^23,25–27^. In our study, left ventricular hypertrophy was determined by measurement of interventricular septal thickness, as this parameter is usually obtained in clinical routine^28^ and multiple studies confirmed that it serves as a reliable surrogate end-point and outcome predictor in cardiovascular disease^29–34^. In this regard, the anti-hypertrophic effect of finasteride in our propensity score matched cohort is comparable to known anti-hypertrophic drugs (such as ACE inhibitors, angiotensin receptor blocker or diuretics)^35–38^. In the light of this evidence, our results provide additional support that the conversion of testosterone to dihydrotestosterone plays an essential role in mediating pathologic left ventricular hypertrophy and that, in turn, the inhibition of this conversion with finasteride might be a possible therapeutic option for the treatment of cardiac hypertrophy and heart failure^16^. This is especially appealing, since finasteride has been in broad clinical use for a number of years and is generally well tolerated, although rare side effects such as sexual dysfunction, depression and high Gleason grade prostate cancer were described^39^. In addition, comprehensive clinical data about finasteride already confirmed its cardiovascular safety^40,41^. Despite this strong evidence of a maladaptive role by androgens on myocardial remodeling^42^, some clinical trials on the other hand showed that testosterone supplementation in patients with chronic heart failure might enhance the patients’ functional capacity or skeletal muscle performance rather than affecting cardiac function or myocardial remodeling^43–49^. A previous study investigated the combined treatment with low-dose testosterone and finasteride in hypogonadal men^50^. In this study, testosterone treatment exerted beneficial effects on skeletal muscle mass, while finasteride co-administration prevented the deleterious impact on other tissues (like the prostate), indicating that not testosterone, but its conversion to the extremely potent dihydrotestosterone is maladaptive.

The main idea of this observational retrospective study was to start to investigate the translational and clinical importance of anti-androgenic therapy with finasteride during heart failure in patients due to the strong preclinical evidence we obtained in mice^16,51^. Consequently, the results from our relative small cohort cannot be extrapolated to general clinical routine, but they might provide a basis for future trials with finasteride as a possible treatment to target cardiac hypertrophy. Alternatively, for the prediction of cardiac therapeutic effects of 5α-reductase inhibitors, Mendelian randomization studies comparing individuals with or without genetic variations (e.g. SNPs) on the 5α-reductase encoding genes (Srd5a1-3) with regard to the development of cardiac hypertrophy and failure could be used to overcome biases related to observational studies^52^.

Some limitations of our study need to be emphasized. First, this is a “real-world” observational retrospective study with an all-comer design (HFpEF or HFrEF patients were eligible). Hence, we did not dissect the intrinsic effects of finasteride treatment on different types of heart failure. Further studies are needed to investigate treatment effects of finasteride in larger cohorts of HFrEF and HFpEF patients separately. Second, our data were derived exclusively from men (mean age about 76 years) and it remains unknown, whether they can be generalized to younger men or women. Third, as with all retrospective observational studies due to their non-randomized nature, unmeasured confounders and missing values may have affected our findings. While the finasteride dosage was known for 141 medical cases, in 23 cases dosages were not documented in the medical records. As exposure, outcome, and confounders are measured simultaneously in cross- sectional studies (like ours), we had no information about the duration of treatment or the duration of disease in our study cohort: finasteride treatment might have been initiated at different stages of the disease and therefore different exposure times may have influenced the results. Hence, we cannot exclude time-related biases, although we find a major distorting effect to be unlikely, as our study cohort equally received guideline-recommended heart failure as well as prostate related medications, indicating comparable disease severity levels. In order to minimize these limitations, a propensity score analysis was employed to balance differences in baseline characteristics between the two groups (accounting for age, cardiovascular risk profile and heart failure medications). In this regard, it is remarkable that the anti-hypertrophic treatment effect of finasteride was detected while the study populations (after propensity score matching) exerted a similar cardiovascular risk profile and equally received guideline-recommended heart failure medications.

## Conclusion

Anti-androgenic therapy with finasteride was associated with attenuated cardiac hypertrophy in patients with heart failure. Therefore, our data encourage further analysis of this approach, for example in larger heart failure patient cohorts or in Mendelian randomization studies.

## Methods

### Study design and setting

This retrospective, cross-sectional single-center study was conducted at Hannover Medical School, a German university hospital. The data of in- and out-patients were obtained by using the medical administrative database for patient documentation. We investigated whether anti-androgenic treatment with finasteride might have beneficial effects on adverse remodeling in patients with heart failure.

### Patient population and data collection

A total of 1654 medical cases (from 1995 to 2015) were identified with documented heart failure, who either received finasteride (with or without tamsulosin) or tamsulosin (only) for an underlying prostate disease. In this “real-world” setting, all-comer patients with diagnosis or criteria for heart failure with reduced ejection fraction (HFrEF; LVEF < 45%) or with preserved ejection fraction (HFpEF; LVEF > 45%) were eligible. Six hundred thirteen patients were excluded from final retrospective analysis because relevant clinical variables for the propensity score model were unavailable (e.g. age, body mass index, systolic blood pressure, diastolic blood pressure, heart rate; all variables included in the propensity score are listed in Table 1). Hence, the final study population comprised 1041 medical cases. Retrospective data review was conducted in accordance with the rules of the local institutional review board (Hannover Medical School) and with permission of the institution’s privacy officer. After consultation with our institution’s ethics committee, approval by this committee and formal consent was not required for this kind of study. All medical cases were identified by using search terms within the medical administrative database with analysis of anonymized data. All data were part of routine diagnosis and treatment. Collected data included demographics, cardiovascular risk factors, cardiac assessments as well as clinical characteristics, vital signs including systolic and diastolic blood pressure, pulse, prescriptions and several laboratory tests, which were all obtained by retrospective chart review. Outcome parameters included cardiac imaging (structure and function), NT-proBNP level and electrocardiogram marker of electrical remodeling (QRS duration, QT and QTc duration), which were collected as part of routine diagnostics by different examinators and obtained by retrospective data review (Table 2). The datasets analysed during the current study are available from the corresponding author on reasonable request and if data privacy permission was given.

### Propensity score methods

Due to the non-randomized nature of a retrospective observational study, a propensity score analysis was performed to yield a balanced distribution of baseline characteristics (including the cardiovascular risk profile) and to estimate finasteride effects on patient outcomes between the treatment and control groups. Briefly, for the final study population a propensity score was calculated using a logistic regression model, in which the treatment exposure (finasteride) was regressed as dependent variable on relevant baseline characteristics. To prevent misspecification of the propensity score model and related biases, it is recommended to include baseline variables related to the outcome^53^, known major risk factors for the outcome^54,55^ and direct causes of the treatment and outcome^56^, while inclusion of colliders or mediators should be avoided^57,58^. Hence, the following baseline variables were included in the propensity score to achieve covariate balance of known major cardiovascular risk factors or confounders of cardiovascular treatment effects: age^59^, diabetes^60^, history of hypercholesterinaemia^52^, hypertension^61^, smoking history^62^, body mass index^63^, COPD^64^, systolic^65^ and diastolic blood pressure^66^, heart rate^67^, ACE inhibitors^68^ or ARB^69^, ß-blocker^70^, MR-antagonists^71^, aspirin^72^, statins^73^. In addition, the underlying prostate disease status was included as it might affect treatment and prognosis of the patients^74,75^. Variables included in the propensity score to achieve covariate balance are listed in Table 1.

Medical cases of treatment and control group were matched on the logit of the estimated propensity scores (1:1 propensity score matching) using calipers width equal to 0.02 of the standard deviation of the logit. While in general, higher caliper widths may result in reduced variance and an increased number of matched subjects, this could on the other hand decrease balance between groups and introduce more bias in estimating treatment effects (trade-off between variance and bias). In our study a lower caliper width (0.02) was therefore used in order to maximize correct matching and to reduce bias; This caliper width has been used by others previously in similar studies^76–78^. Ongoing research addresses the choice of optimal caliper width during propensity score based matching: one study proposed to use a caliper width equal to 0.2 of the standard deviation of the logit of the propensity score, which may need to be taken into account when interpreting our results^79^. Absolute standardized difference ≤0.1 for measured covariates suggested appropriate balance between the groups (Table 1 and Fig. S1 in the Data Supplement).

### Descriptive statistics

All data were analysed using SPSS 24 for Windows (IBM SPSS statistics). All graphs were compiled with the use of Prism 7 software (GraphPad). Continuous variables are presented as means and standard deviations (SD). Analysis of data distribution was performed with the Kolmogorov–Smirnov and Shapiro-Wilk-Test. Categorical variables are provided with absolute numbers (*n*) and percentages (%). We used the students T-test or Mann Whitney U test (when appropriate) to compare continuous variables and the Pearson chi-square test to compare categorical variables. Spearman’s rank correlation coefficient was analysed to evaluate possible correlation between two variables. The null hypothesis was tested against a two-sided alternative hypothesis at a significance level of 5%. As our study is the first study to start investigating whether the results from our previous preclinical study in mice might be also translated to patients, this exploratory study was designed to investigate primarily left ventricular hypertrophy and additional preplanned outcome variables associated with maladaptive cardiac remodeling (Table 2). For this type of explorative study adjustment for multiple comparisons is not desirable and not recommended^80–82^, because the chance that effective treatment effects of finasteride are not discovered (type II errors) increases, although without mathematical correction for multiple comparisons the risk of type I errors in non-primary outcomes increases (result of false significance) which may need to be taken into account interpreting the results. Additional studies are needed to confirm the results derived from our exploratory study.

## Supplementary information

Data Supplement
