# Supplementary material for: Anti-androgenic therapy with finasteride in patients with chronic heart failure - a retrospective propensity score based analysis
Source: Sci Rep. 2019 Jul 12;9:10139. doi: 10.1038/s41598-019-46640-8 (PMC6626053; doi:10.1038/s41598-019-46640-8)
Supplement: Supplementary file 1 — Data Supplement [file 41598_2019_46640_MOESM1_ESM.pdf]

Supplementary information

Anti-androgenic therapy with finasteride in patients with chronic heart failure - a retrospective propensity score based analysis

Badder Kattih[a,b,§,#], Lukas Simon Elling[a,#], Christel Weiss[c], Marieke Bea[a], Carolin Zwadlo[a], Udo Bavendiek[a], Johann Bauersachs[a] and Joerg Heineke[a,b,*]

[a] Department of Cardiology and Angiology, Hannover Medical School, Carl-Neuberg-Straße 1, 30625 Hannover, Germany.

[b] Department of Cardiovascular Research, European Center for Angioscience (ECAS), Medical Faculty Mannheim of Heidelberg University, University Medical Centre Mannheim, Ludolf-Krehl Straße 7-11, 68167 Mannheim, Germany.

[c] Department for Medical Statistics and Biomathematics, Medical Faculty Mannheim of Heidelberg University, University Medical Centre Mannheim, Ludolf-Krehl Straße 9-13, 68167 Mannheim, Germany.

[§] Co-Correspondence: Department of Cardiovascular Research, European Center for Angioscience (ECAS), Medical Faculty Mannheim of Heidelberg University, University Medical Center Mannheim, Ludolf-Krehl Straße 7-11, phone no.: +49211-38371860, 68167 Mannheim, Germany. Electronic address: Badder.Kattih@medma.uni-heidelberg.de

[*] Correspondence: Department of Cardiovascular Research, European Center for Angioscience (ECAS), Medical Faculty Mannheim of Heidelberg University, University Medical Center Mannheim, Ludolf-Krehl Strasse 7-11, phone no.: +49211-38371850, 68167 Mannheim, Germany. Electronic address: Joerg.Heineke@medma.uni-heidelberg.de

[#] BK and LSE contributed equally to this work.

Supplemental Figure S1

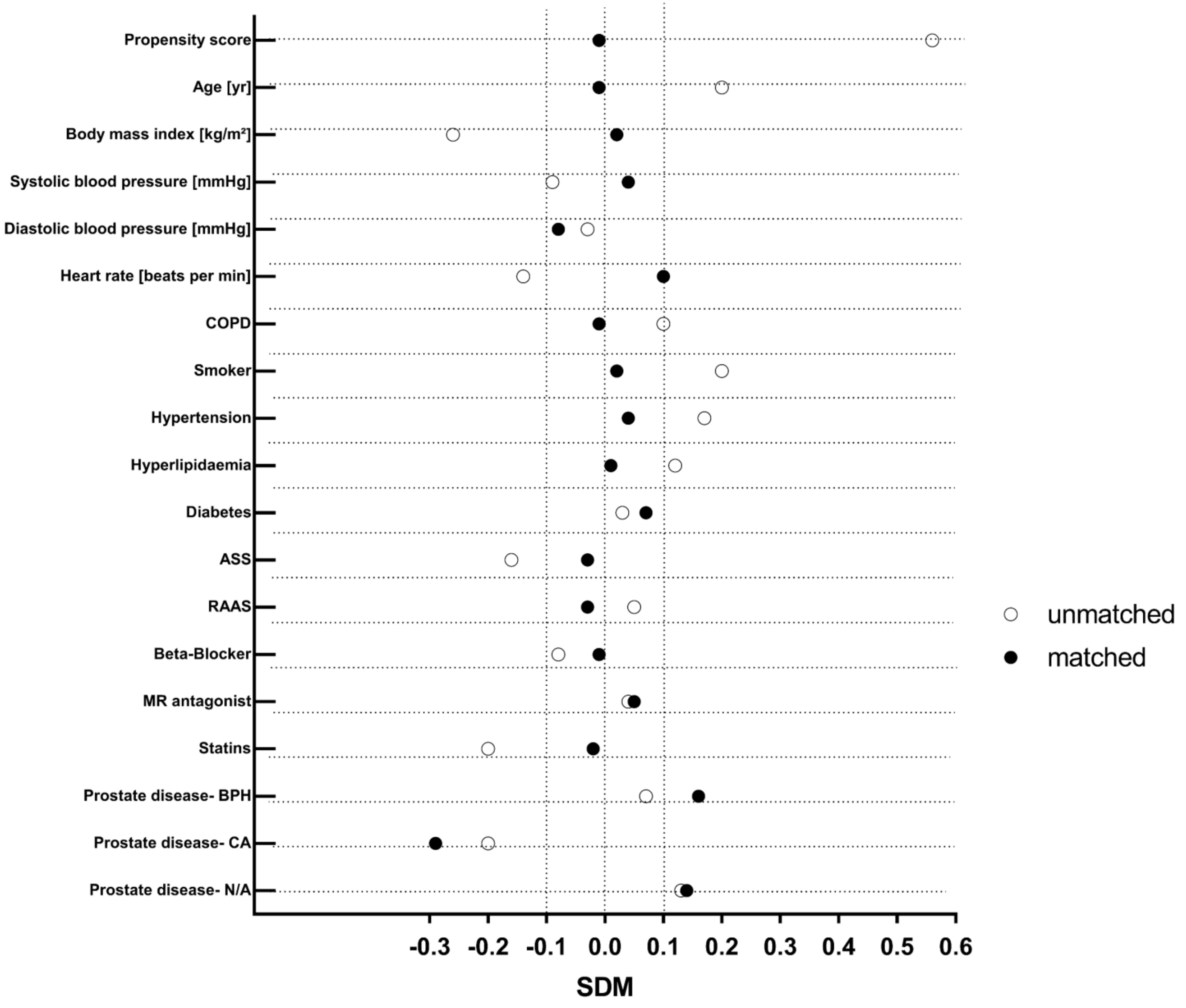

Supplemental Figure S1. Comparison of covariate balance by using absolute standardized differences between treated and control subjects in the unmatched (white circles) and the matched study sample (black circles). The standardized difference is the difference of the mean values or proportions (treatment - control group) divided by the pooled standard deviation. It measures the effect size between two groups and is independent from sample sizes. Matching on the logit of the propensity score has diminished many of systematic differences between treated and untreated subjects and resulted in balance in measured variables.

Supplementary Table S1. Baseline characteristics pre- and post-propensity score matching.

(detailed version of Table 1)

	unmatched cohort (total n = 1041)			matched cohort (total n = 328)		
	**control group (total n = 868)**	**treatment group (total n = 173)**	**p value**	**control group (total n = 164)**	**treatment group (total n = 164)**	**p value**
**Propensity score**	0.153924± 0.0964985	0.227713± 0.113189	<0.001	0.227713± 0.102900	0.217158± 0.102488	0.996
**Logit of propensity score**	-1.911635± 0.889430	-1.338785± 0.690426	<0.001	-1.393076± 0.657590	-1.393104± 0.655751	0.996
**Male sex**	868/868 (100.0)	173/173 (100.0)		164/164 (100.0)	164/164 (100.0)	
**Finasteride**	0/868 (0.0)	173/173 (100.0)		0/164 (0.0)	164/164 (100.0)	
**Finasteride dose [mg/d]**	0.0±0.0 (0/868)	5.1±0.7 (150/173)		0.0±0.0 (0/164)	5.1±0.6 (141/164)	
**Tamusulosin**	868/868 (100.0)	74/173 (42.8)	<0.001	164/164 (100.0)	72/164 (43.9)	<0.001
**Age [yr]**	74.1± 8.0 (n=868)	75.9± 6.7 (n=173)	0.006	75.9± 7.7 (n=164)	75.8± 6.7 (n=164)	0.862
**Body mass index [kg/m²]**	27.1± 3.8 (n=868)	25.8± 3.8 (n=173)	<0.001	25.9± 3.6 (n=164)	25.9± 3.8 (n=164)	0.746
**Body surface area [m^2]**	1.99± 0.18 (868)	1.96± 0.16 (173)	0.064	1.93± 0.16 (164)	1.96± 0.16 (164)	0.106
**Systolic blood pressure [mmHg]**	132.4± 46.3 (n=868)	129.1± 19.6 (n=173)	0.352	128.6± 21.6 (n=164)	129.5± 18.9 (n=164)	0.607
**Diastolic blood pressure [mmHg]**	74.5± 11.7 (n=868)	74.1± 12.3 (n=173)	0.576	75.0± 11.9 (n=164)	73.9± 11.6 (n=164)	0.346
**Heart rate [beats per min]**	72.6± 17.1 (n=868)	70.2± 13.3 (n=173)	0.209	68.3± 15.7 (n=164)	70.1± 13.5 (n=164)	0.152
**COPD**	129/868 (14.9)	34/173 (19.7)	0.113	33/164 (20.1)	32/164 (19.5)	0.890
**History of smoking**	291/868 (33.5)	79/173 (45.7)	0.002	72/164 (43.9)	74/164 (45.1)	0.824
**Hypertension**	656/868 (75.6)	145/173 (83.8)	0.019	134/164 (81.7)	137/164 (83.5)	0.662
**Hyperlipidemia**	529/868 (60.9)	117/173 (67.6)	0.098	108/164 (65.9)	109/164 (66.5)	0.907
**Diabetes mellitus**	240/868 (27.6)	51/173 (29.5)	0.624	42/164 (25.6)	48/164 (29.3)	0.458
**Aspirin**	485/868 (55.9)	80/173 (46.2)	0.020	80/164 (48.8)	77/164 (46.9)	0.740
**ACEi/ARBs**	670/868 (77.2)	138/173 (79.8)	0.457	134/164 (81.7)	132/164 (80.5)	0.778
**Beta-blockers**	661/868 (76.2)	124/173 (71.7)	0.212	120/164 (73.2)	119/164 (72.6)	0.901
**MR-antagonists**	159/868 (18.3)	35/173 (20.2)	0.555	29/164 (17.7)	33/164 (20.1)	0.573
**Statins**	611/868 (70.4)	101/173 (58.4)	0.002	101/164 (61.6)	99/164 (60.4)	0.821
**Diuretics**	571/868 (65.8)	108/173 (62.4)	0.397	115/164 (70.1)	103/164 (62.8)	0.160
**Prostate disease**						
**Benign prostatic hyperplasia**	836/868 (96.3)	169/173 (97.7)	<0.001	154/164 (93.9)	160/164 (97.6)	0.005
**Prostate cancer**	32/868 (3.7)	1/173 (0.6)		10/164 (6.1)	1/164 (0.6)	
**N/A**	0/868 (0.0)	3/173 (1.7)		0/164 (0.0)	3/164 (1.8)	
**NYHA status**						
**NYHA I**	293/868 (33.8)	37/173 (21.4)	<0.001	50/164 (30.5)	32/164 (19.5)	0.045
**NYHA II**	269/868 (31.0)	43/173 (24.9)		51/164 (31.1)	43/164 (26.2)	
**NYHA III**	235/868 (27.1)	73/173 (42.2)		50/164 (30.5)	71/164 (43.3)	
**NYHA IV**	36/868 (4.1)	8/173 (4.6)		7/164 (4.3)	7/164 (4.3)	
**N/A**	35/868 (4.0)	12/173 (6.9)		6/164 (3.7)	11/164 (6.7)	
**Acute decompensation**	88/868 (10.1)	28/173 (16.2)	0.021	16/164 (9.8)	26/164 (15.9)	0.098
**Valvular disease**	137/868 (15.8)	28/173 (16.2)	0.895	30/164 (18.3)	27/164 (16.5)	0.662
**Diastolic dysfunction**	228/868 (26.3)	38/173 (21.9)	0.236	38/164 (23.2)	37/164 (22.6)	0.895

Values are expressed as mean ± SD or n/ total n (%). Abbreviation: ACEi= angiotensin converting enzyme inhibitor, ARB= angiotensin receptor blocker, COPD= Chronic obstructive pulmonary disease, N/A = not available, NYHA= New York heart association.

Supplementary Table S2. Outcome parameters pre- and post- propensity score matching normalized to BSA.

	unmatched cohort (total n=1041)				matched cohort (total n=328)			
	control group (total n= 868)	treatment group (total n= 173)	p value	ATT (95% confidence intervall)	control group (total n= 164)	treatment group (total n= 164)	p value	ATT (95% confidence intervall)
**normalized to BSA**								
**IVS [mm]**	6.5± 1.3 (n=464)	6.6±1.3 (n=122)	0.451	0.1 (-0.2 to 0.3)	6.9±1.4 (n=93)	6.5±1.3 (n=114)	0.029	-0.4 (-0.8 to -0.04)
**LVEDD [mm]**	27.0± 4.6 (n=540)	28.0±4.5 (n=141)	0.007	0.9 (0.2 to 1.8)	27.3±4.8 (n=109)	28.1±4.5 (132)	0.171	0.8 (-0.4 to 2.0)
**LA-PLAX [mm]**	22.8± 4.1 (n=487)	23.2±4.5 (n=140)	0.346	0.4 (-0.4 to 1.2)	23.3±4.2 (n=96)	23.1±4.4 (n=131)	0.670	-0.2 (-1.4 to 0.9)

Abbreviation: ATT= average treatment effect on the treated, BSA= body surface area, IVS= Interventricular septum, LA-PLAX= Left atrium in parasternal long axis, LVEDD= Left ventricular enddiastolic diameter.

Supplementary Table S3. Distribution of patients per severity level of interventricular septal thickness.[17]

	matched cohort	
	**control group**	**treatment group**
**Normal range [6-10 mm]**	7/23 (30.4)	16/23 (69.6)
**Mildly abnormal [11-13 mm]**	46/107 (43.0)	61/107 (57.0)
**Moderatly abnormal [14-16 mm]**	31/64 (48.4)	33/64 (51.6)
**Severe abnormal [>16 mm]**	9/13 (69.2)	4/13 (30.8)

Values are expressed as n/ total n (%). Raw values of Figure 2B.

Supplementary Table S4. Left ventricular cardiac function in the unmatched and matched study cohort.

	**unmatched cohort (total n=1041)**	**matched cohort (total n=328)**
**Left ventricular function**		
**normal [>55%]**	397/1041 (38.1)	118/328 (36.0)
**mildly reduced [45-55%]**	282/1041 (27.1)	79/328 (24.1)
**moderately reduced [35-44%]**	238/1041 (22.9)	81/328 (24.7)
**severly reduced [<35%]**	124/1041 (11.9)	50/328 (15.2)

Values are expressed as n/ total n (%). Raw values of Figure 1.

Supplementary Table S5. Distribution and level of NT-proBNP stratified by decompensation status.

	unmatched cohort (total n=1041)				matched cohort (total n=328)			
	control group (total n= 868)	treatment group (total n= 173)	p value	ATT (95% confidence intervall)	control group (total n= 164)	treatment group (total n= 164)	p value	ATT (95% confidence intervall)
**(Ln)NT-proBNP (no acute decompensation)**	6.6±1.6 (127/780)	6.4±1.7 (28/145)	0.757	-0.2 (-0.9 to 0.4)	7.1±1.7 (28/148)	6.3±1.7 (27/138)	0.079	- 0.8 (-1.7 to 0.1)
**(Ln)NT-proBNP (acute decompensation)**	8.7±1.1 (36/88)	8.1±0.7 (13/28)	0.103	-0.7 (-1.2 to -0.1)	9.5±1.1 (6/16)	8.0±0.7 (12/26)	0.003	-1.5 (-2.4 to -0.6)
